# Examination of Speech Analysis to Predict Suicidal Behavior in Depression

**DOI:** 10.1192/j.eurpsy.2024.167

**Published:** 2024-08-27

**Authors:** S. Yünden, M. Ak, M. Sert, S. Gica, O. Çinar, Y. A. Acar

**Affiliations:** ^1^Psychiatry, Necmettin Erbakan University Meram Faculty of Medicine Hospital, Konya; ^2^Computer Engineering, Başkent University Faculty of Engineering; ^3^Emergency Service, Acıbadem Ankara Hospital, Ankara, Türkiye; ^4^Emergency Service, Mercer University School of Medicine, Macon, United States

## Abstract

**Introduction:**

Suicide is one of the leading causes of preventable deaths worldwide. The psychiatric disorder that is most strongly associated with suicide is depression. It is crucial to develop clinical tools that can provide objective data to assess suicide risk in clinical settings. Depression and high suicide risk may lead to physiological changes that can affect the speech pattern. Prior research has indicated that the acoustic and prosodic characteristics of speech may hold potential clues for assessing suicide risk. Additionally, specific speech parameters may serve as discriminators for identifying individuals at risk. In recent years, deep learning-based models have yielded successful results in identifying such alterations in speech signals.

**Objectives:**

The aim of our study was to examine specific voice analysis parameters between control, depressive and high suicide risk groups. We also aimed to investigate the effect of voice-related variables in predicting suicidal behavior in patients with depression using an artificial intelligence model. The results of voice analysis are intended to serve as a starting point for the development of future artificial intelligence algorithms.

**Methods:**

The study sample consisted of 30 near-term suicidal patients, 30 patients with major depression and 30 healthy controls. The participants were presented with a pre-determined text and a voice recording was carried out. Feature extraction and model training for three tasks, namely depression or not, suicide or not, and depression or suicide were carried out. Mel-Frequency Cepstral Coefficients (MFCCs), deep learning-based (VGGish), formant and prosodic features were extracted to analyze the sound characteristics of the participants. The Support Vector Machine was used as the machine learning algorithm for classification and the three models were trained for each task. A 10-fold cross-validation was carried out and presented by metrics including accuracy, precision, sensitivity and specificity.

**Results:**

Among the metrics examined, MFCCs for the “Suicide or not” task were found to be more successful with rates of 0.90, 0.88, 0.93 and 0.86 for accuracy, precision, sensitivity, and specificity, respectively. MFCCs were also more successful for the “Depression or suicide” task with rates of 0.68, 0.66, 0.76, and 0.60 for accuracy, precision, sensitivity, and specificity, respectively. Among the metrics examined for the “Depressed or not” task, VGGish was more successful with rates of 0.73, 0.81, 0.70, and 0.76 for accuracy, precision, sensitivity, and specificity, respectively.

**Conclusions:**

To the best of our knowledge, our study is the first to compare the VGGish and other features of speech (MFCCs, prosodic, formant features) between high suicide risk, depression and control groups. Classification parameters developed using the VGGish and MFCCs features of speech could be useful in predicting suicide risk in future studies.

**Disclosure of Interest:**

None Declared

